# Mind the Decimal Point: A Case of Diazoxide Overdose-Induced Ileus

**DOI:** 10.7759/cureus.62088

**Published:** 2024-06-10

**Authors:** Mostafa M Meleis, Putt P Vithayaveroj, Natalie E Ebeling-Koning, John D DelBianco, Ryan M Surmaitis

**Affiliations:** 1 Department of Emergency and Hospital Medicine, Lehigh Valley Health Network/University of South Florida (USF) Morsani College of Medicine, Allentown, USA

**Keywords:** diazoxide, overdose, tenfold dosing error, ileus, preterm infant, hyperinsulinism

## Abstract

Diazoxide is the only medication approved by the United States Food and Drug Administration for the treatment of hyperinsulinism-induced hypoglycemia. Overdose is infrequently reported. This case describes a preterm four-week-old male who was prescribed diazoxide and chlorothiazide for perinatal stress-induced hyperinsulinism. The patient presented to the emergency department with feeding intolerance and abdominal distension following an accidental 10-fold diazoxide overdose. On presentation, vital signs were remarkable for tachycardia and intermittent tachypnea. Physical exam revealed a grossly distended abdomen. Laboratory abnormalities included a glucose of 216 mg/dL, sodium of 132 mmol/L, and chloride of 98 mmol/L. Abdominal X-ray interpretation found moderate gaseous distension suggestive of generalized ileus. The patient was admitted to the neonatal intensive care unit (NICU), and a nasogastric tube was placed. He received intravenous dextrose fluids, and enteral feeds were resumed as serial X-rays showed interval improvement. The patient remained in the NICU for several days to monitor bowel movements and resolution of ileus and he was discharged after improvement. While diazoxide overdose is rarely reported, and ileus due to such is documented even less frequently, 10-fold medication dose errors are common among infants. The source of the 10-fold mistake is often decimal points, leading zeros, or trailing zeros. Utilizing the smallest possible syringe for the prescribed dose may reduce the incidence of medication errors.

## Introduction

Newborns frequently experience hypoglycemia, but typically glucose stabilizes within a few days [1]. When hypoglycemia is severe and persistent, hyperinsulinism is the most common cause. Hyperinsulinism can be hereditary, or it can be caused by risk factors including perinatal asphyxia, maternal diabetes mellitus, and intra-uterine growth restriction [2]. Rapid diagnosis is crucial, as hyperinsulinism in newborns is a major cause of hypoglycemia-associated brain injury and neurocognitive delays [1,3]. Diagnostic criteria include hypoglycemia (blood glucose less than 50 mg/dL), detectable serum insulin level, low ketones, and low free fatty acids [4]. Diazoxide is the only medication approved by the United States Food and Drug Administration for the treatment of hyperinsulinism-induced hypoglycemia, and its use has risen over the past decade [5,6]. Diazoxide stimulates the sulfonylurea receptor 1 subunit of adenosine triphosphate-sensitive potassium (K-ATP) channels on pancreatic beta cells, resulting in increased potassium efflux, cell hyperpolarization, reduced calcium influx, and thus suppression of calcium-mediated insulin secretion [5,7,8]. Potential adverse effects include edema, heart failure, pulmonary hypertension, ileus, necrotizing enterocolitis, vomiting, diabetic ketoacidosis, hyperuricemia, thrombocytopenia, and neutropenia [3,7,9-12]. Overdose is infrequently reported [13]. We report a case of a preterm four-week-old male who developed hyperglycemia and ileus after an accidental 10-fold diazoxide overdose.

This case in part was previously presented as an abstract at the American College of Medical Toxicology Annual Meeting (April 12, 2024, Washington, DC, United States), and the Pennsylvania College of Emergency Physicians Scientific Assembly (May 2, 2024, Pocono Manor, PA, United States).

## Case presentation

A four-week-old male, born at 32 weeks gestation due to placental insufficiency and growth restriction, was prescribed diazoxide (8 mg/kg/day divided every 12 hours) and chlorothiazide (10 mg/kg/day divided every 12 hours) for perinatal stress-induced hyperinsulinism. This was the initial dose as prescribed in the neonatal intensive care unit (NICU) and was not titrated or altered by the time of discharge. On the day of the patient’s discharge from the NICU, he was given his appropriate morning dose of 50 mg/mL oral diazoxide in the hospital; however, later that evening, his mother administered 1.4 mL instead of 0.14 mL of diazoxide, resulting in a 10-fold overdose. The patient became fussy, fed poorly overnight, and was noted to have an elevated morning blood sugar of 200 mg/dL. Upon realizing her dosing error, the patient’s mother brought him to the emergency department (ED). The prescription bottle was not brought with the patient to the ED; however, the prescription instructions were confirmed with the patient’s mother to have been correct as electronically prescribed.

**Figure 1:**
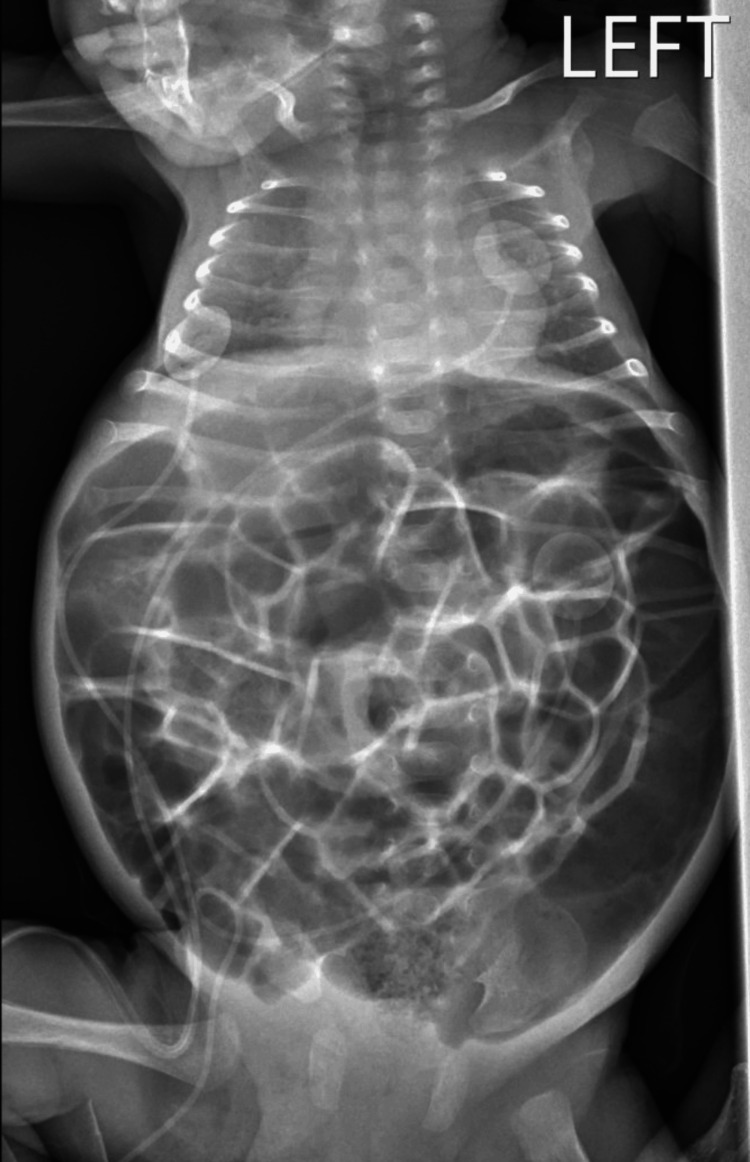
Supine anteroposterior radiograph of chest and abdomen demonstrating moderate gaseous distension suggestive of generalized ileus

The patient presented to the ED with feeding intolerance and abdominal distension. His initial vital signs were blood pressure 78/40 mmHg, heart rate 167 beats per minute, temperature 97.6 degrees F, respiratory rate 46, and oxygen saturation 99% on room air. A physical exam revealed a grossly distended abdomen with decreased bowel sounds. Abnormal labs included a glucose of 216 mg/dL, sodium of 132 mmol/L, and chloride of 98 mmol/L. Other notable normal values included beta-hydroxybutyrate of 0.27 mmol/L, serum bicarbonate of 26 mmol/L, anion gap of 8, and negative acetone. Venous blood gas testing was also within normal range. No lactic acid level was drawn. The chest and abdominal X-ray interpretation found moderate gaseous distension suggestive of generalized ileus (Figure 1). Notably, there was no evidence of pulmonary edema.

**Figure 2:**
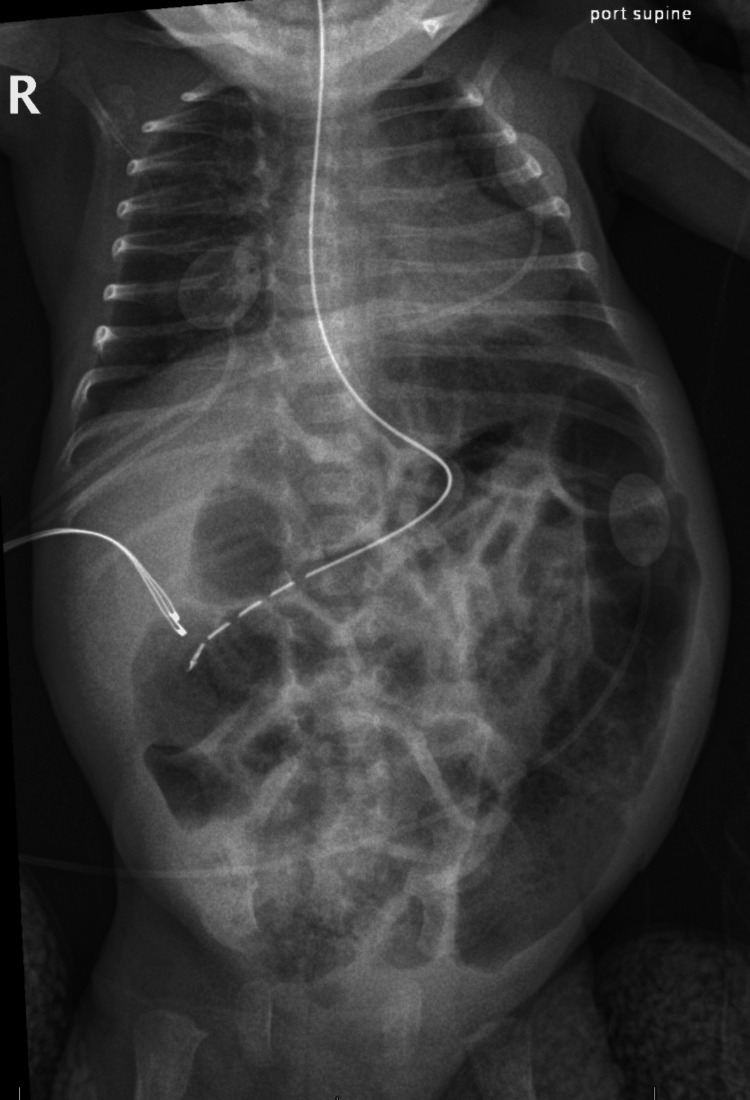
Supine anteroposterior radiograph of chest and abdomen demonstrating interval improvement of bowel gas pattern

The patient was admitted to the NICU, and a nasogastric tube was placed. Cardiac monitoring and continuous pulse oximetry were maintained and remained normal throughout the admission. He received supportive care in the form of bowel rest and intravenous dextrose-containing fluids. Enteral feeds were resumed 11 hours later, as serial X-rays showed interval improvement of bowel gas pattern (Figure 2). No insulin infusion was required. Diazoxide was resumed by the NICU the day after admission, approximately 30 hours after the overdose, and at that time the blood glucose was 97 mg/dL. The patient remained in the NICU for several days to monitor bowel movements and resolution of ileus, and after improvement of symptoms he was discharged from the hospital on day four.

## Discussion

Diazoxide overdose is rarely reported, and we identified a single other case report referencing supratherapeutic ingestion of diazoxide where the patient manifested with diabetic ketoacidosis. The specific dose was not included in the case report [13]. Ileus is mentioned frequently as a possible adverse effect of diazoxide, but we identified only one report detailing a patient who experienced such. Reference is made to this patient who experienced ileus while on a therapeutic dose of diazoxide within an article detailing the management and outcome of patients with congenital hyperinsulinism [12]. We hypothesize the mechanism of ileus in patients receiving diazoxide may be secondary to retained fluid, as well as gastrointestinal smooth muscle relaxation, due to its mechanism of action on K-ATP channels. There are also various other more well-known side effects that may present with initiation of or during treatment with diazoxide, most notably edema, heart failure, pulmonary hypertension, ileus, necrotizing enterocolitis, diabetic ketoacidosis, hyperuricemia, thrombocytopenia, and neutropenia [3,7,9-12]. The frequency at which patients experience edema while on diazoxide often leads to the concurrent initiation of diazoxide and a prophylactic diuretic [5].

While diazoxide overdoses are uncommon, a more broadly applicable takeaway from this case is that medication dosing errors in infants, in particular 10-fold dose errors, are unfortunately very common. The source of the 10-fold mistake is often decimal points, leading zeros, or trailing zeros [14]. Further challenges can arise with patients requiring sub-milliliter doses of medications, as the smallest commonly available syringe is a one-milliliter syringe. Utilizing the smallest possible syringe for the prescribed dose may reduce the incidence of medication errors.

Additionally, more advanced prophylactic caregiver education can also prevent medication dosing errors. Advanced counseling strategies for caregivers including demonstration, drawings/pictures, and teach-back/show-back have been associated with reduced caregiver dosing errors [15]. These strategies were used to educate the mother after admission for overdose; however, only verbal understanding of the plan of care was documented upon initial discharge from the NICU. Standard NICU discharge at our institution includes reviewing medications with caregivers. Providing routine, advanced, preventative counseling to caregivers, including practicing syringe use and demonstrating proficiency in medication administration, may help prevent future medication errors.

## Conclusions

Diazoxide overdose is rare and seldom reported in current literature, though this could be due to underreporting. Abdominal distention and ileus are less common but may be the presenting symptoms of toxicity. While ileus is listed as a possible side effect of diazoxide, there is minimal literature documenting specific cases of this adverse effect. Infants are at particularly high risk for medication dosing errors. Caregiver-advanced counseling strategies should be utilized routinely and prophylactically, and the use of the smallest possible syringes should be employed to reduce the risk of dosage errors in infants discharged on medications.
